# Adapting an intervention to support young caregivers of cancer survivors: A study protocol

**DOI:** 10.1371/journal.pone.0284896

**Published:** 2023-04-27

**Authors:** Janet Njelesani, Melinda S. Kavanaugh, Jean Hunleth

**Affiliations:** 1 Department of Occupational Therapy, New York University, New York, New York, United States of America; 2 Helen Bader School of Social Welfare, University of Wisconsin – Milwaukee, Milwaukee, Wisconsin, United States of America; 3 Division of Public Health Sciences, Washington University School of Medicine in St. Louis, St. Louis, Missouri, United States of America; Tokyo Medical and Dental University: Tokyo Ika Shika Daigaku, JAPAN

## Abstract

**Introduction:**

Of the family members providing care, in the United States over 5.4 million are young people (<18 years of age) and they are the caregivers receiving the least support overall. Given the need to support cancer survivors through a family-centered practice approach, this lack of support and intervention for young caregivers represents a substantial gap in cancer care. In this study, we will adapt a young caregivers intervention, YCare, with young caregivers in families affected by cancer in order to advance support for families in cancer settings. YCare is an intervention that improves the support young caregivers provide through a peer-engaged, multidisciplinary model but has previously not been studied in the cancer care setting.

**Methods:**

Guided by the updated Consolidated Framework for Implementation Research (CFIR) we will engage stakeholders (i.e., young caregivers, cancer survivors, health care providers) using qualitative (i.e., one-on-one semi-structured interviews) and arts-based methods. Stakeholders will be recruited via cancer registries and community partners. Data will be analyzed descriptively using deductive (e.g., CFIR domains) and inductive (e.g., cancer practice settings) approaches.

**Discussion:**

The results will indicate the critical components for adapting the YCare intervention to the cancer practice context including new intervention elements and key characteristics. Adapting YCare to a cancer context will address a critical cancer disparity issue.

## Introduction

Cancer survivorship, the time between diagnosis and end-of-life, is rising in the United States, with more than 1.8 million adult diagnoses in 2020 [1]. Cancer is also a main health condition for which people receive family care [2]. Cancer caregiving is considered different from other chronic conditions; caregivers spend more hours per day caring, leading to greater distress and financial strain [3]. Cancer survivors may rely on their children to provide the care, particularly in the absence of another adult in the home (i.e., not residing; out at work). In the United States, more than 5.4 million young people under age 18 are estimated to provide care to persons with impairments and/or health conditions [2]. These young people are carrying out caregiving tasks without support, training, or acknowledgment from health service providers.

Health service providers play a role in cancer care given the physical, emotional, and cognitive implications associated with chemotherapy, radiation, and surgery. Health service providers thus need to be equipped with and use evidence-based interventions not just to support cancer survivors, but also family members who are providing care. However, a substantial gap in cancer practice exists. This critical gap in service includes the lack of provision of a holistic family-centered practice approach to meet the changing needs of the cancer survivor and their caregivers, while taking into account that young caregiver’s roles in cancer caregiving. Our study seeks to close the gap by identifying needs and adapting an intervention for young caregivers.

YCare is an intervention that improves the support young caregivers provide. Guided by the tenets of Individual and Family Self-Management Theory, YCare includes modules on basic care, feeding, assistive devices, and caregiver support [4]. YCare is delivered by a trained multidisciplinary team including physical therapy occupational therapy, respiratory therapy, speech language pathology and social work, and uses a structured teach-back method to instill confidence in carrying out the activities. The teach-back follows specific steps: therapists talk about the task and ask whether youth have participated in the task with their family member; show how to do the task; have youth try the task; and ask the youth to teach back the task to the group. At the end of each module, a debriefing session occurs wherein youth identify what tasks they liked and felt confident about, and what tasks still made them uncomfortable. YCare also provides social support to address the burden of caregiving on young people’s engagement in other activities including education, play, and leisure and a handbook for each participant to guide the sessions. Each module is delivered in small peer groups of 4–6 participants of similar ages (i.e., <13 years, ≥13 years) and lasts 50 minutes. YCare was developed and tested for feasibility by a multidisciplinary team of health providers, including physical therapy, speech-language pathology, occupational therapy, and social work [4]. YCare has shown significant positive outcomes in families with neurological disease [5], yet given it’s modular nature, was created to allow for adaption across other illness and injury populations. Thus, the contribution of the proposed study will be the adaptation of the YCare intervention to cancer caregiving.

### Study framework

The updated Consolidated Framework for Implementation Research (CFIR) will be used to guide the study, as it is a widely used framework aimed at adapting an intervention to a new site [6]. CFIR best fits our adaptation needs because of its attention to inner (i.e., Siteman Cancer Center (SCC) delivery of care) and outer (i.e., St. Louis context) setting. By identifying setting specific barriers and facilitators through CFIR grounded methods, we will be able to rigorously adapt the intervention.

### Aim

The aim of this study is to adapt YCare to a cancer care setting.

## Methodology

### Setting

Siteman Cancer Center in St. Louis was selected as the study site because it is a National Cancer Institute (NCI) designated cancer center with an exceptional rating from the NCI. SCC achieved that rating for its cutting-edge research and care available to a large catchment area, and its commitment to reducing cancer disparities through community-based research and outreach.

### Timeline

The study will be conducted in phases. Phase 1 (months 1–8) is the elicitation phase when data will be collected and analyzed. In Phase 2 (months 9–12) the intervention will be adapted for a cancer setting.

### Participants

Proposed participants include: 1) health service providers who have experience in cancer practice settings, ≥19 years old, and English or Spanish speaking, 2) cancer survivors who are ≥19 years old, English or Spanish speaking, diagnosed with any type of cancer and treated at SCC in the past 5 years, have a child <19 years of age, and experience financial strain, 3) youth participants who are13-18 years of age, English or Spanish speaking, identify as providing care to an adult who is eligible for the study as noted above. To focus on economic disparities, participants will be from households experiencing financial strain, identified using a screener tool that measures financial strain and that includes one self-reported financial status question (i.e., In general, how do you find your household’s finances usually work out at the end of the month? Is there; not enough to make ends meet, enough to make ends meet, or plenty of money left over?) [7]. People who reply “not enough to make ends meet” will be eligible to participate in the study.

We will access the SCC cancer registry data to identify cancer survivors. Adults meeting the inclusion criteria will be mailed a letter and recruitment materials, as well as recruitment materials to share with a child who provides care. We will follow up the mailing with a recruitment phone call to answer any questions, verify eligibility, and obtain contact information for the caregiver. Caregivers will also complete a brief eligibility screening over the phone. Those interested and eligible will be scheduled to participate. Purposive sampling will be used to select and invite cancer survivors and caregivers of varying genders, ages, races, and ethnicities to achieve a balance of representation and to increase the depth of understanding across people who may hold different views [8]. The form of purposive sampling to be used will be quota sampling, which will include recruiting a minimum number of participants from each category. Specifically we will aim to recruit a minimum of five cancer survivors each who are female, under 50 years of age, and identify as a person from a historically underrepresented group. We will aim to recruit a minimum of five caregivers each who are female, below 15 years of age, and identify as a person from a historically underrepresented group. Health service providers will be recruited through recruitment details sent through their manager.

SCC is the only NCI-designated cancer center in the region, treating over 9000 people annually; thus, recruitment is expected to be feasible. Qualitative data will be generated with approximately 45 participants, including young caregivers (n = 15), adult cancer survivors (n = 15), and health service providers (n = 15). The sample size will be increased should data saturation not be reached.

### Data collection

Semi-structured interviews will be carried out with each participant group. Each individual data collection session will take 60 minutes. Interview questions will be developed from the relevant CFIR domains (e.g., local conditions, compatibility, mission alignment), using the updated CFIR, and also focus on perceptions and informational needs of caregivers, strengths, YCare content, strategies for increasing participation in YCare, and cultural appropriateness of YCare materials. Interviews will be audio-recorded and transcribed. A tailored semi-structured interview guide will be developed for each participant group (see Table 1 for examples of interview questions for each group).

**Table 1:** Stakeholder interview questions.

Stakeholder group	Example interview questions
Cancer survivors	Can you tell me what kinds of things [young caregiver] has done to help or care for you since your diagnosis? What other assistance or help do you think would benefit families with young people in similar situations? What topics do you think are most important to include in YCare to help the young people?
Young caregivers	During visits to health care providers, did they ever talk about your role in the care? Can you tell me about any help you received that made things easier for you to care for your parent during their cancer diagnosis and treatment? what types of things would you include in YCare?
Health service providers	What kind of interventions or formal/informal support are you currently providing to caregivers of cancer survivors in your oncology practice setting? How will the infrastructure of your organization affect the implementation of the YCare intervention? What kinds of changes will be needed to accommodate the intervention?

For youth, in addition to the interviews, arts-based participatory techniques previously developed by the co-PIs in research with youth, including young caregivers, will also be used [9–12]. These methods will include warm-up activities, writing, drawing, and photo elicitation, and are considered best practice in research with young people [13, 14]. For youth, because of the additional data collection methods used, two data collection sessions will occur. The data collection methods will be first piloted with a member of each group to ensure the activities can be completed in no more than 60 minute sessions.

### Data analysis

Qualitative software (NVivo 11.0^®^) will be used to facilitate content analysis and will include deductive (e.g., CFIR domains) and inductive (e.g., cancer practice settings) approaches. Data will be coded using deductive and inductive codes by two coders. Rigor will be achieved through analysis meetings to review the codes and make suggestions about modifying the codes, or adding more inductive codes as needed [15]. The YCare intervention is currently focused on supporting young caregivers for people living for neurological disease and will be adapted to the needs of cancer survivors. We will therefore adapt YCare using findings from the empirical data (e.g., CFIR implementation content will guide how the adapted YCare should be planned; oncology practice content will guide the caregiving activities to be included in the adapted module) so that the content and modes of delivery, etc. are appropriate for cancer survivors treated at SCC. Throughout analysis to ensure trustworthiness of the qualitative data we will create a comprehensive audit trail that clearly documents what we did and why we did it at each stage of the study with enough detail for others to replicate the work.

### Ethical considerations

Approval to conduct this study has been obtained by New York University (IRB-FY2022-6586) and Washington University in St. Louis (IRB ID #: 202205008). Minimal risk to participants is expected and informed written consent and written assent will be obtained. For youth participants, written consent will be obtained from parents. Informed consent materials will specify the study details, including the purpose of the study, a description of the study methods and what will be expected of the subject, the risks and benefits of participation, and contact information for reporting adverse events.

## Discussion

Findings of this study will lead to the adaptation of the YCare intervention for a cancer setting. Following adaptation, a study will be conducted to test the effectiveness of the adapted intervention with caregivers of cancer survivors. Our long-term goal is to strengthen services for young caregivers and their families, an area where the implementation of evidence-based interventions is urgently needed.

We recognize that only recruiting English or Spanish speaking participants and recruiting from one cancer center are limitations of the study design that make the work less generalizable and exclude a greater diversity of participants. However, given the small sample size and study resources, we aim to balance an increase in heterogeneity among participants with the feasibility of carrying out the study. Furthermore, given the demographic characteristics of communities in which the study is located, the English or Spanish speaking requirement should not exclude a large number of people and will not compromise our ability to recruit historically underrepresented groups.

Results will be published in peer-reviewed journals and presented at conferences focusing on cancer care and health service delivery. Furthermore, knowledge mobilization activities will occur with cancer survivors and their families.
